# First report of *Diomusguilavoguii* Duverger, 1994 (Coleoptera, Coccinellidae, Diomini) predating on papaya mealybug *Paracoccusmarginatus* from China

**DOI:** 10.3897/BDJ.11.e113291

**Published:** 2023-11-16

**Authors:** Jiamin Zhuang, Lizhi Huo, Mingjie Tang, Xiufeng Xie, Xiaosheng Chen

**Affiliations:** 1 Department of Forest Protection, College of Forestry and Landscape Architecture, South China Agricultural University, Guangzhou 510642, China Department of Forest Protection, College of Forestry and Landscape Architecture, South China Agricultural University Guangzhou 510642 China; 2 Engineering Research Center of Biological Control, Ministry of Education, Guangzhou 510642, China Engineering Research Center of Biological Control, Ministry of Education Guangzhou 510642 China; 3 Guangzhou Collaborative Innovation Center on Science-Tech of Ecology and Landscape, Guangzhou Institute of Forestry and Landscape Architecture, Guangzhou 510419, China Guangzhou Collaborative Innovation Center on Science-Tech of Ecology and Landscape, Guangzhou Institute of Forestry and Landscape Architecture Guangzhou 510419 China; 4 Guangdong Agriculture Industry Business Polytechnic College, Guangzhou 510507, China Guangdong Agriculture Industry Business Polytechnic College Guangzhou 510507 China

**Keywords:** Coccinellidae, new record, larva, pupa, *Diomushennessyi*

## Abstract

**Background:**

*Diomusguilavoguii* Duverger, 1994, an adventive species, is recorded from Guangzhou (Guangdong Province), China for the first time. Larvae of *D.guilavoguii* were collected in association with an invasive mealybug, *Paracoccusmarginatus* Williams & Granara de Willink, 1992, infesting papayas, cassava and several ornamental plants. However, little has been known about the biology of *D.guilavoguii*, especially the morphology of their larvae since their original descriptions.

**New information:**

*Diomusguilavoguii* Duverger, 1994, native to Conakry, Guinea (Africa), is recorded as established in Guangdong Province for the first time. However, it is unclear when and how *D.guilavoguii* spread from Africa to Guangzhou, Guangdong Province. Both the adult and larva feed on the invasive mealybug *Paracoccusmarginatus* Williams & Granara de Willink (Hemiptera, Pseudococcidae) that infests papaya and ornamental plants. In this paper, the external morphology and male genitalia of adults are re-described. The detailed descriptions of larva and pupa are also provided for the first time. The status of *D.guilavoguii* and *D.hennessyi* Fürsch, 1987 are discussed.

## Introduction

*Diomus* was originally established by Mulsant (1850) as a subgenus of *Scymnus* Kugelann, 1794, based on unique abdominal postcoxal lines that meets the posterior margin of the first abdominal ventrite. Weise (1895) first elevated *Diomus* to generic status, based on the median fusion of the first and second abdominal ventrite and the peculiar form of the abdominal postcoxal lines. Since then, Diomus has been treated either as a subgenus of Scymnus or a valid genus by various researchers. Gordon (1999) proposed the tribe Diomini to include *Diomus* and four other Neotropical genera according to a comprehensive study of South American Coccinellidae. Ślipiński (2007) recorded the genera *Diomus*, *Dichaina* Weise, 1923 and proposed a new genus *Andrzej* Ślipiński in this tribe from Australia.

*Diomusguilavoguii* Duverger, 1994 was described from Guinea by Duverger (1994) and not was discovered in other regions. According to our observation, *D.guilavoguii* preys mainly on the invasive mealybug, *Paracoccusmarginatus* Williams & Granara de Willink, 1992 (Hemiptera, Pseudococcidae), which infested with papaya and many different kinds of ornamental plants, such as *Mussaendapubescens* W. T. Aiton, Barbados nut (*Jatrophacurcas* L.) and Spicy Satropha (*Jatrophaintegerrima* Jacq.). Some of these host plants are imported from abroad. It is obvious that *D.guilavoguii*, as a natural enemy of *P.marginatus*, has great potential for biological control.

The papaya mealybug, *P.marginatus* is a globally invasive pest that causes significant yield losses in various crops (Mani et al. 2012). Since its original description in 1992, *P.marginatus* as a native species of Mexico and Central America has spread rapidly to many other countries including USA, Africa and Asia (Miller and Williams 1999, Ahmed et al. 2015, Finch et al. 2021). It was recorded from China for the first time in 2013 in Guangdong and Yunnan Provinces (Ahmed et al. 2015). The papaya mealybug not only directly affects plant growth by sucking sap, but also indirectly affects the development of sooty mould on plants by excreting honeydew, hindering photosynthesis and gas exchange (Kirsur et al. 2014, Ahmed et al. 2015).

Although the papaya mealybug is native to Mexico and Central America, it is not a pest there due to the presence of its natural enemies keeping it under control. However, when it invaded other countries or regions, it was considered a serious pest mainly due to the lack of natural predators (Ahmed et al. 2015). Saengyot and Burikam (2011) carried out field surveys of host plants in Thailand to investigate the host plants and natural predators of the papaya mealybug. Their investigation indicated that 11 species of natural enemies controlling the papaya mealybug’s populations either by parasitism or predation, including five coccinellids, *Cryptogonusorbiculus* (Gyllenhal, 1808), *Sasajiscymnusquinquepunctatus* (Weise, 1923), Scymnus (Pullus) quadrillum Motschulsky, 1858, *Scymnus* sp. and *Stethorus* sp. (Saengyot and Burikam 2011). Mani et al. (2012) mentioned that a total of 22 natural enemies were reported on papaya mealybug in different countries. Amongst them, eight species belonged to ladybird beetles (Coleoptera, Coccinellidae), such as *Cryptolaemusmontrouzieri* Mulsant, 1853, *Nephusbilucernarius* (Mulsant, 1850), *Scymnustaiwanus* (Ohta, 1929) (= Scymnus (Pullus) quadrillum, Motschulsky, 1858), *Brumoidessuturalis* (Fabricius, 1798), *Hyperaspissilvestrii* Weise, 1909, *Cheilomenessexmaculata* (Fabricius, 1781), *Coccinellatransversalis* Fabricius, 1781 and *Chilocorusnigritus* (Fabricius, 1798). These ladybird beetles are playing a key role in biological control of the papaya mealybug.

In this paper, *Diomusguilavoguii* Duverger, 1994 is recorded as being well established in Guangzhou for the first time. Detailed descriptions and illustrations of the adult, pupa and larvae are provided. Diagnostic characters for the genus and species are also given.

## Materials and methods

Specimens examined were collected from Guangdong Province, China and deposited in the Department of Entomology, South China Agricultural University, Guangzhou, China (SCAU). The terminology used in the descriptions of larva follows Kamiya (1965) and Gordon and Vandenberg (1991) and the descriptions of adults follow Ślipiński (2007) and Ślipiński and Tomaszewska (2010).

Measurements were taken using a micrometer attached to a SteREO Discovery V20 dissecting stereoscope and are defined as follows: (TW) total width, across both elytra at widest part; (TH) total height, at highest part of elytra in lateral view; (TL) total length, from apical margin of clypeus to apex of elytra; (PL) pronotal length, from the middle of anterior margin to the base of pronotum; (PW) pronotal width at widest part; (EW) elytral width, equal to TW; (EL) elytral length, along suture from base to apex including scutellum; (HW) head width, at widest part including eyes.

Male and female genitalia were dissected, cleared in a 10% solution of sodium hydroxide (NaOH) by boiling for several minutes and placed on slides for further study. Photographs of the adult were taken with a digital camera (EOS 5D Mark IV, Canon) and photographs of their genitalia were taken using digital cameras (ZEISS Imager M2 and Axiocam 506 Color) attached to the microscope.

**Figure 1.**
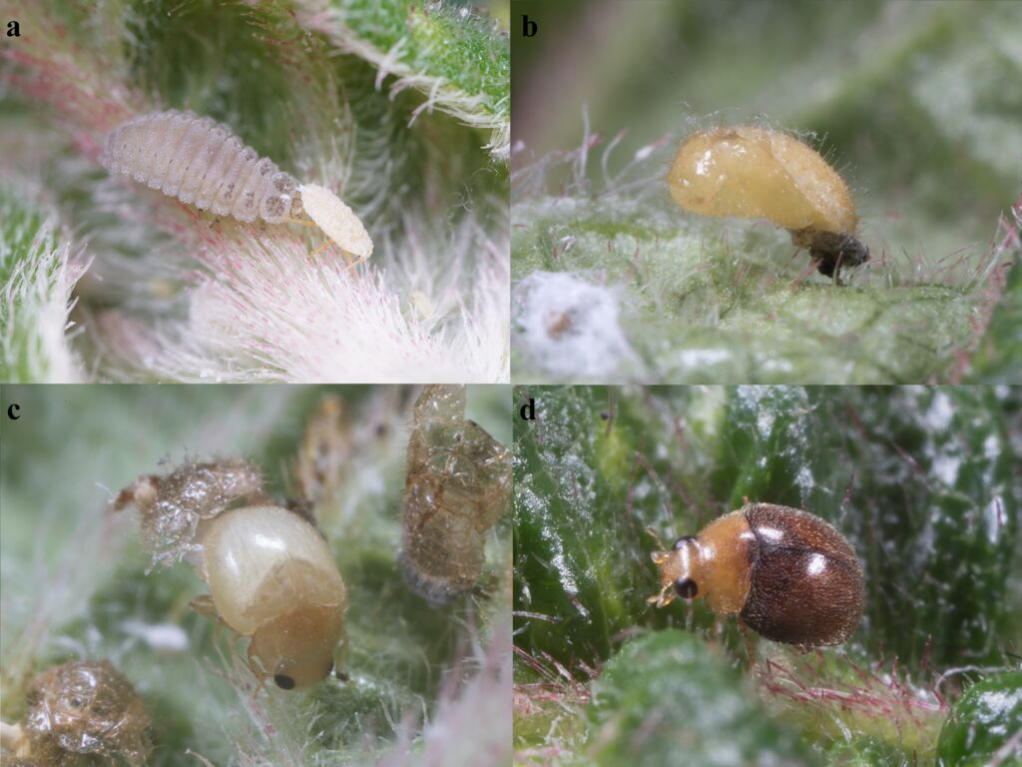
Life stages of *Diomusguilavoguii* Duverger, 1994. **a** larva, fourth instar; **b** pupa; **c** adult, newly emerged; **d** adult.

Larvae were reared in 500 ml plastic dishes in rearing chambers at 25 ± 1℃, 70% ± 10% R.H. and 12:12 h L:D. The food, *Paracoccusmarginatus*, was supplied daily to maintain the population stock (Fig. 1).

For morphological studies, the larvae were soaked in 75% alcohol for conservation. The larvae were photographed using digital cameras (EOS 5D Mark IV, Canon), attached to a focus stacking rail (WeMacro Rail). The software Helicon Remote and Helicon Focus were used to capture and render images respectively from the camera. Mouthparts, head and tarsal claw of the larvae were dissected, cleared in a 10% NaOH solution, boiled for half an hour, washed in distilled water and placed on slides. Colour images were captured with digital cameras (ZEISS Imager M2 and Axiocam 506 Color) attached to a dissecting microscope using ZEN 2.3 software. All photographs were edited using Adobe Photoshop CC 2018 and Adobe Illustrator 2020.

## Taxon treatments

### Diomus

Musant, 1850

79F536A9-6B44-5792-ABB6-CCEE21B29A7F

Scymnus (Diomus) Mulsant, 1850: 951. Type species: *Coccinellathoracica* Fabricius, 1801, by subsequent designation by Korschefsky (1931).
*Diomus*: -Weise (1895: 144); Gordon (1976: 319); Gordon (1999: 13); Pang and Ślipiński (2009: 646).
Nephus (Diomus) by Iablokoff-Khnzorian (1976: 377) (Iablokoff-Khnzorian 1976).
*Amidellus* Weise, 1923 - Weise (1923: 141) (Weise 1923). Type species: *Scymnusementitor* Blackburn, 1895 by original designation. Synonymised by Ślipiński (2007: 87).

#### Diagnosis

**Figure 2.**
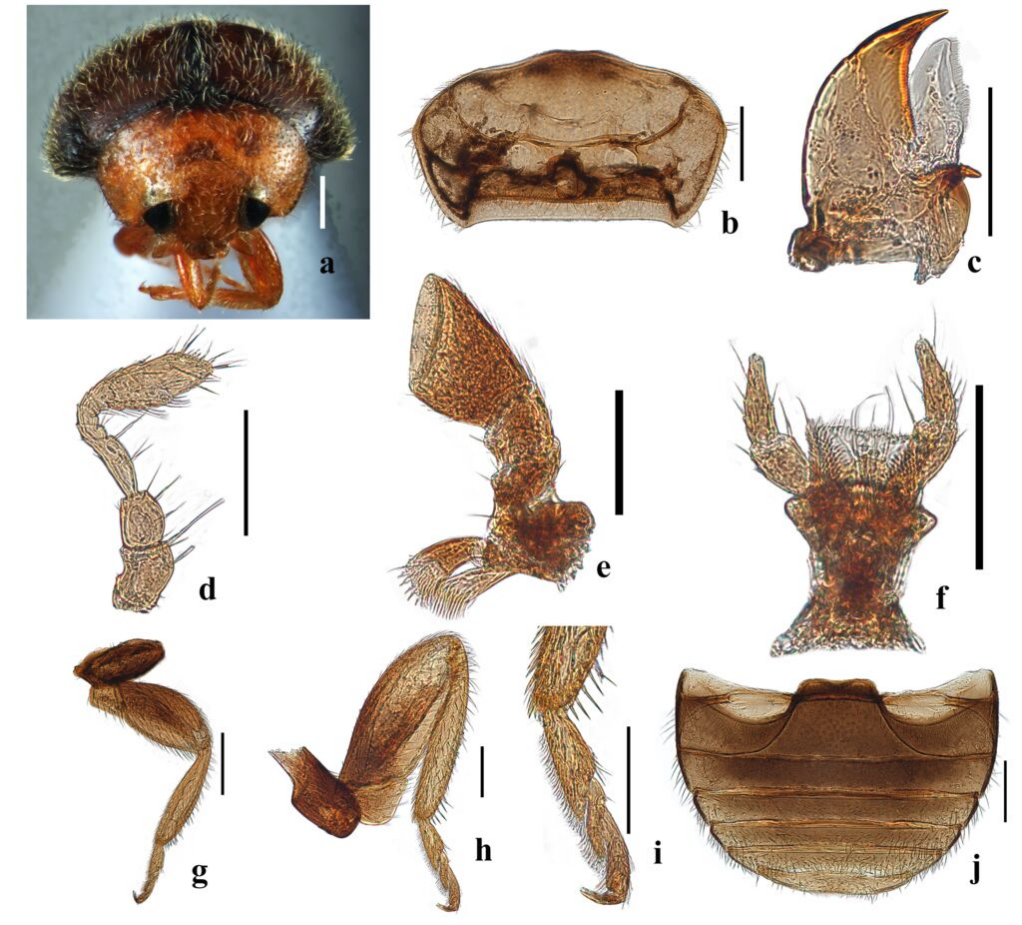
Main characters of *Diomusguilavoguii* Duverger, 1994, adult: **a** adult in front view; **b** prothorax, ventral view; **c** mandible; **d** antenna; **e** maxilla; **f** labium; **g** hind leg; **h** front leg; **i** tarsus of front leg; **j** abdomen in ventral view. Scale bars: a = 0.2 mm; b, g, j = 200 μm; c, d, e, f, h, i = 50 µm.

This genus can be separated from other genera within the tribe Diomini by the following characters: antennae composed of 11 or 10 antennomeres; slightly shorter than head capsule with pedicel narrower than scape; antennomere 3 elongate; antennal club indistinct and multi-segmented (Fig. 2d). Abdominal postcoxal lines merging with hind margin of ventrite (Fig. 2j). For detailed descriptions, see Gordon (1976), Gordon (1999) and Pang and Ślipiński (2009).

#### Distribution

Worldwide, but certainly more diverse in the Southern Hemisphere like Neotropical and Australian Regions (Pang and Ślipiński 2009).

#### Biology

Most species of the genus have a wide range of host preferences that mainly feed on mealybugs (Fig. 1a), aphids, scale insects and whiteflies. Some species were introduced into various regions for the purpose of biological control. For instance, *Diomuspumilio* Weise, 1885, originally from Australia, was introduced to California to manage the Albizzia psyllid, *Psyllauncatoides* (Ferris & Klyver, 1932) (=*Acizziauncatoides* (Ferris & Klyver, 1932)), on Acacia (Gordon and Hilburn 1990).

### Diomus guilavoguii

Duverger, 1994

2EE9704D-61C3-5868-A504-DF0942303C13

*Diomusguilavoguii* Duverger, 1994: 121.

#### Materials

- **Type status:** Other material. **Occurrence:** recordedBy: Jiamin Zhuang; individualID: SCAU (E) 17572; individualCount: 1; sex: male; lifeStage: adult; behavior: running; occurrenceID: 414A66E3-1DDC-5F13-A1DC-68F59BDD3139; **Taxon:** scientificName: *Diomusguilavoguii*; class: Insecta; order: Coleoptera; family: Coccinellidae; genus: Diomus; **Location:** country: China; countryCode: CHN; stateProvince: Guangdong; municipality: Guangzhou; locality: South China National Botanical Garden; verbatimElevation: 47.9 m; decimalLatitude: 23.180592; decimalLongitude: 113.366531; **Identification:** identifiedBy: Xiaosheng Chen; dateIdentified: 10-12-2022; identificationReferences: Duverger 1994; **Event:** samplingProtocol: observe; year: 2022; month: 9; day: 2; **Record Level:** institutionID: South China Agricultural University; institutionCode: SCAU; basisOfRecord: Preserved Specimen
- **Type status:** Other material. **Occurrence:** recordedBy: Jiamin Zhuang; individualID: SCAU (E) 17573; individualCount: 1; sex: female; lifeStage: adult; occurrenceID: 660BFF3A-1B43-5FF4-8786-652803487BBC; **Taxon:** scientificName: *Diomusguilavoguii*; class: Insecta; order: Coleoptera; family: Coccinellidae; **Location:** country: China; countryCode: CHN; stateProvince: Guangdong; municipality: Guangzhou; locality: South China National Botanical Garden; verbatimElevation: 47.9 m; decimalLatitude: 23.180592; decimalLongitude: 113.366531; **Identification:** identifiedBy: Xiaosheng Chen; dateIdentified: 10-12-2022; identificationReferences: Duverger 1994; **Event:** samplingProtocol: observe; year: 2022; month: 9; day: 23; **Record Level:** institutionID: South China Agricultural University; institutionCode: SCAU; basisOfRecord: Preserved Specimen
- **Type status:** Other material. **Occurrence:** recordedBy: Mingjie Tang; individualCount: 13; sex: 6 male, 2 female, 9 unsexed specimens; lifeStage: 8 adult, 4 pupa, 5 larvae; occurrenceID: 5D4D546E-BD3F-5241-A8E4-A4CA13770E31; **Taxon:** scientificName: *Diomusguilavoguii*; class: Insecta; order:  Coleoptera; family: Coccinellidae; **Location:** country: China; countryCode: CHN; stateProvince: Guangdong; municipality: Guangzhou; locality: Campus of South China Agricultural University; verbatimElevation: 33.4 m; decimalLatitude: 23.162782; decimalLongitude: 113.355362; **Identification:** identifiedBy: Xiaosheng Chen; dateIdentified: 10-12-2022; identificationReferences: Duverger 1994; **Event:** samplingProtocol: observe; year: 2022; month: 9; day: 27; **Record Level:** institutionID: South China Agricultural University; institutionCode: SCAU; basisOfRecord: Preserved Specimen
- **Type status:** Other material. **Occurrence:** recordedBy: Xiufeng Xie; individualCount: 10; sex: 9 male, 1 female; lifeStage: adult; occurrenceID: 297D0151-D750-534B-AA7A-BB0B3C8411CA; **Taxon:** scientificName: *Diomusguilavoguii*; class: Insecta; order: Coleoptera; family: Coccinellidae; **Location:** country: China; countryCode: CHN; stateProvince: Guangdong; municipality: Guangzhou; locality: Campus of Guangdong AIB Polytechnic College; verbatimElevation: 22.5 m; decimalLatitude: 23.284552; decimalLongitude: 113.612518; **Identification:** identifiedBy: Xiaosheng Chen; dateIdentified: 07-23-2023; identificationReferences: Duverger 1994; **Event:** samplingProtocol: observe; year: 2023; month: 7; day: 2; **Record Level:** institutionID: South China Agricultural University; institutionCode: SCAU; basisOfRecord: Preserved Specimen

#### Description

**Adult**. TL: 1.53-1.54 mm, TW: 1.16-1.21 mm, TH: 0.57-0.63 mm, TL/TW: 1.27-1.32, PL/PW: 0.31-0.32, EL/EW: 1.00-1.03, HW/PW: 0.61-0.65, PW/EW: 0.73.

**Figure 3.**
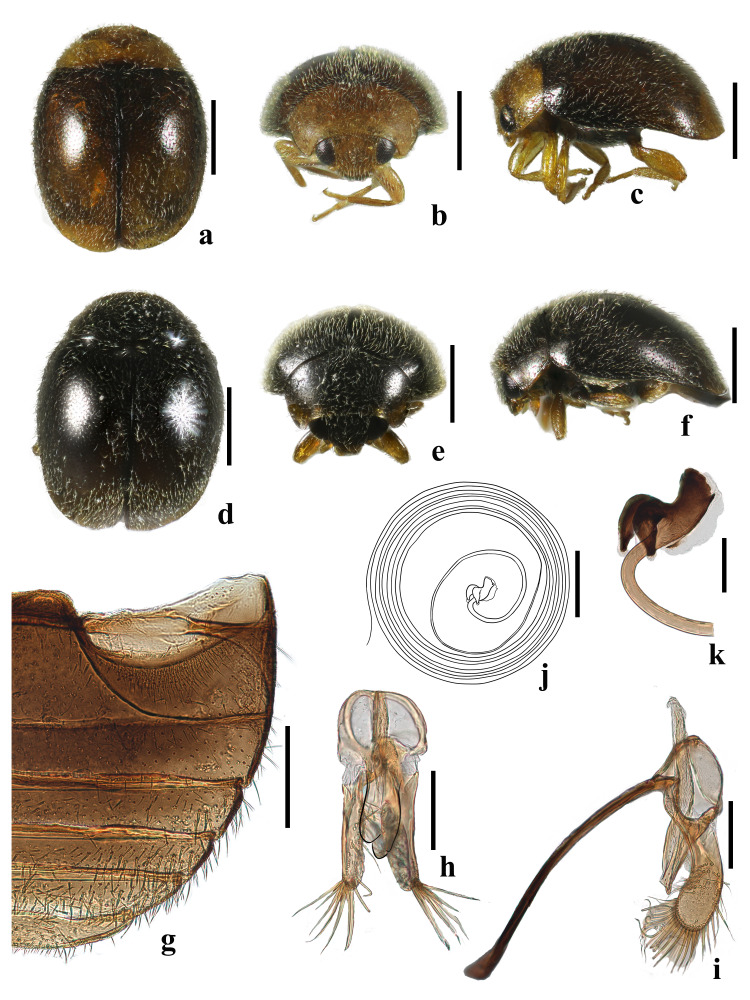
Adult of *Diomusguilavoguii* Duverger, 1994. **a** male, dorsal view; **b** male, front view; **c** male, lateral view; **d** female, dorsal view; **e** female, front view; **f** female, lateral view; **g** abdomen; **h** tegmen, inner view; **i** tegmen, lateral view; **j** penis; **k** penis capsule. Scale bars: a-f = 0.5 mm; g, j = 200 μm; h-k = 100 μm. Black lines: outlines of apical penis guide.

**Male**: Body oval, weakly convex; usually winged; dorsum uniformly hairy. Head transverse, dorsally not covered by pronotum (Fig. 2a). Winged. Head yellowish-brown; elytra brownish, with the apex yellowish. Dorsum evenly covered by dense, whitish pubescence with hairs not forming distinct patterns, but mostly pointing posteriorly (Fig. 3a-c). Eye very large, finely facetted, weakly emarginated and moderately separate on vertex; inner orbits converging anteriorly; frons twice width of eye (Fig. 3b). Antennae of the type specimens are composed of 11 antennomeres, whereas those from China have only 10 antennomeres; antennomere 3 about 2.2 times as long as 4; antennal club consisting of 4 terminal antennomeres; terminal antennomeres much larger than its preceding 3 antennomeres, with apical margin strongly obliquely truncate (Fig. 2d). Mandibles bifid apically, with well-developed molar tooth (Fig. 2c). Terminal maxillary palpomere at least weakly expanded apically (Fig. 2e). Mentum (Fig. 2f) subtrapezoidal, broadest anteriorly and medially shallowly rounded. Labial palps with three palpomeres, terminal labial palpomere subcylindrical, shorter than penultimate one (Fig. 2f). Prosternal process 0.9 times width of coxal cavity; prosternal carinae incomplete anteriorly; surface between carinae punctate and setose (Fig. 2b). Elytral epipleuron narrow, incomplete apically, not foveate (Fig. 3a and d). Pronotal disc evenly convex. Prosternum moderately long in front of coxae, arcuate (Fig. 2b); prosternal process broad, usually with complete carinae, rarely without carinae. Anterior margin of mesoventrite straight medially. Mesoventrite slightly narrower than coxal diameter with metaventrite always projected forward and arcuate; metaventral postcoxal lines strongly recurved. Tibial spurs absent (Fig. 2g-i).

**Male genitalia**: Penis slender, extremely long (Fig. 3j). Penis capsule highly sclerotised, inner arm bifurcate, abruptly recurved (Fig. 3k). Penis guide with short asymmetrical apical tooth in inner view (Fig. 3h); in lateral view, penis guide widest at base, then tapering gradually to a blunt apex and bearing a horned projection at the proximal end (Fig. 3i). Parameres stout, distinctly longer than penis guide in lateral view, widened and apically rounded, densely setose apically (Fig. 3i). Tegminal strut slender, distinctly longer than combined length of phallobase and penis guide (Fig. 3h and i).

**Female**: Externally identical to male, but head black and elytra black with the apex more or less finely yellowish (Fig. 3d-f). Prothorax black with lateral and anterior margins brown (Fig. 3d-f).

**Figure 4.**
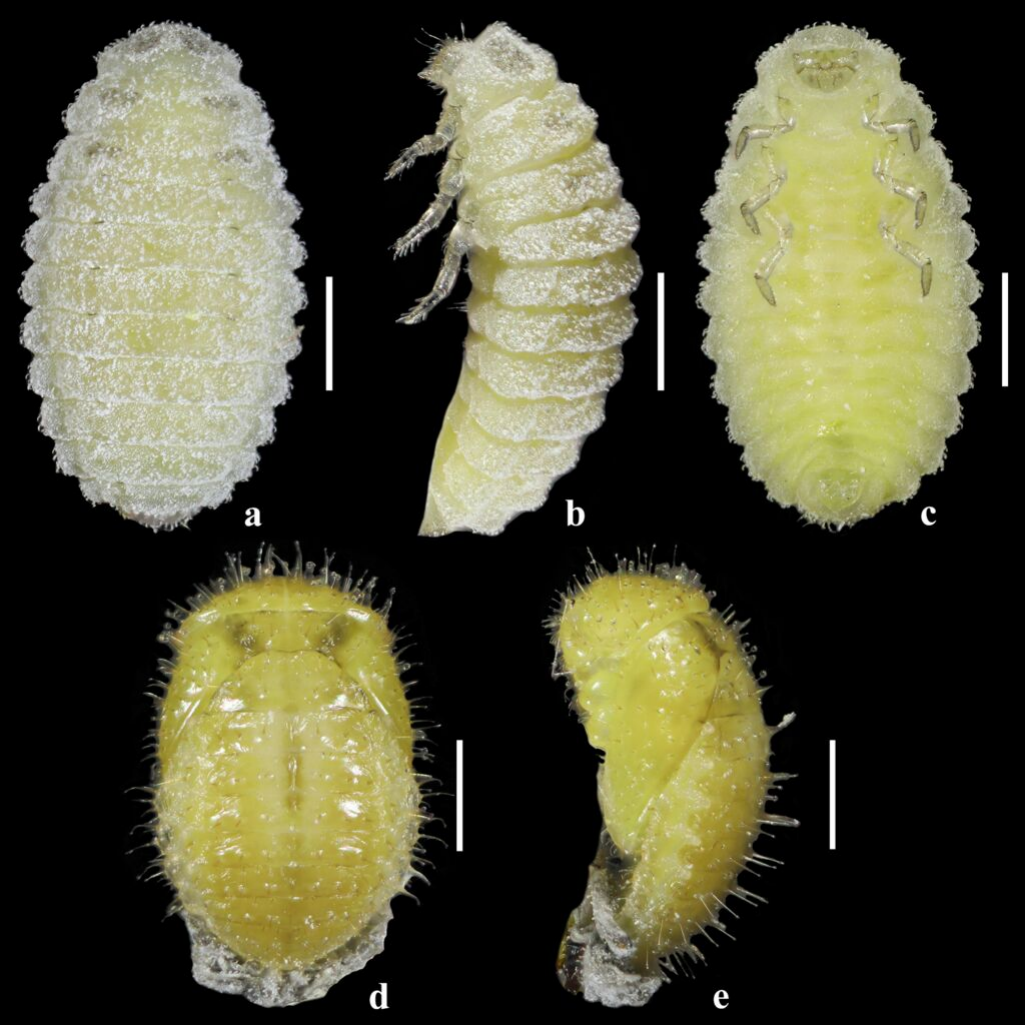
Larva and pupa of *Diomusguilavoguii* Duverger, 1994. **a** fourth instar larva, dorsal view; **b** fourth instar larva, lateral view; **c** fourth instar larva, ventral view; **d** pupa, dorsal view; **e** pupa, lateral view. Scale bars: 0.5 mm.

**Figure 5.**
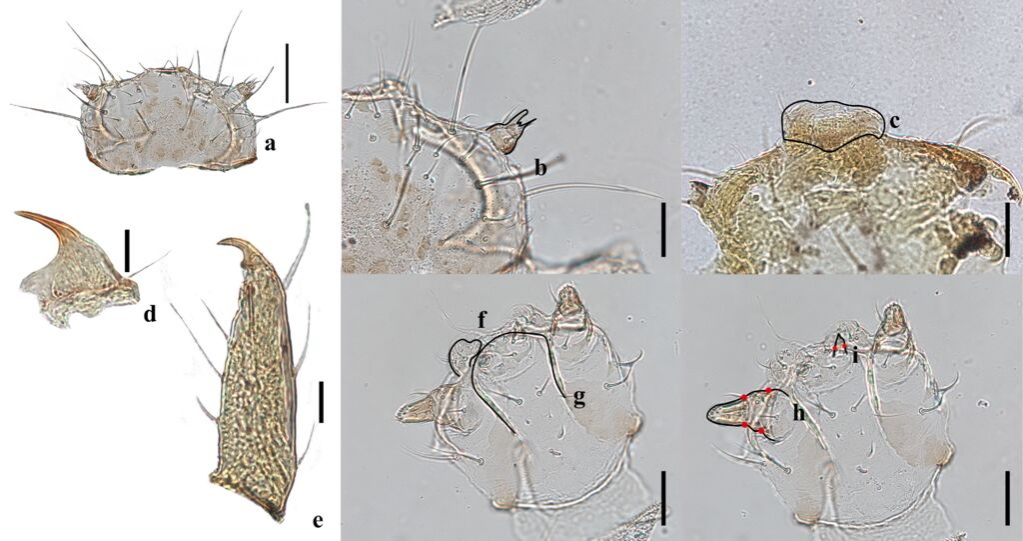
Larval characters of *Diomusguilavoguii* Duverger, 1994, fourth instar larva; **a** head; **b** antenna; **c** labrum; **d** mandible; **e** tarsal claw; **f** maxilla; **g** labium; **h** maxillary palp; **i** labial palp. Scale bars: a = 100 µm; b, c, f, g, h, i = 50 µm; d, e = 25 μm. Black lines: outlines of structures; red dots: boundaries of segmentation of maxillary palp and labial palp.

**Four instar larva** (Figs 4, 5). Length 2.27 mm; width 1.28 mm. Body elongate oval, with short bristles and waxy, light yellow, with black stripes (Fig. 4a-c). Head: light yellow, nearly semi-round (Fig. 5a). Epicranial suture with frontal arms indistinct (Fig. 5a). Three hemispherical stemmata dark, arranged in triangle, near the base of antennae. Antennae composed of only one antennomere with a long bristle and apical papillae (Fig. 5b). Labrum squarish, with sparse bristles (Fig. 5c). Mandible sclerotised with one apical tooth and a long bristle above the condyle, without basal tooth (Fig. 5d). Maxillary palp with three palpomeres and apex with sensillae (Fig. 5h). Labium with spare and thin bristles, labium palp with two palpomeres and small stout sensillae at apex (Fig. 5g, i). Thorax: pronotum light yellow, has spare bristles with black stripes on two sides and two semi-round dorsal plates. Meso- and metanotum light yellow, with black stripes on two sides (Fig. 4a-c). Legs light yellow, short, with sparse pale yellow hairs; tarsal claw without basal tooth and with a long lateral bristle on the external face of the tooth and spare bristles on the internal face of the tibiae (Fig. 5e). Abdomen: nine segments, light yellow with spare tortuous bristles (Fig. 4a-c).

**Pupa** (Fig. 4d-e). Length 1.93 mm; width 1.25 mm. Body oval, yellow, with black stripes and bristles. Thorax: pronotum and metanotum yellow, trailing edge black; mesonotum yellow, with black stripes on two sides; metanotum yellow, nearly triangular. Abdomen: seven segments visible.

#### Diagnosis

This species is similar to most members of the genus *Diomus* in general habitus and colour pattern, but can be distinguished from those species by the extremely long penis and the robust penis capsule with a bifurcate inner arm.

#### Distribution

Guinea (Conakry), China (Guangdong) **new record**.

## Discussion

*Diomusguilavoguii* is recorded from China for the first time. The specimens examined in the present paper show some minor variation in antennae however, their external appearance of adult and male genitalia were in agreement with the detailed descriptions and illustrations given by Duverger (1994). Interestingly, the antennae of the specimens collected from Guangzhou have only 10 antennomeres, while the type specimens described by Duverger (1994) have 11 antennomeres. This phenomenon is also present in some species of the genus *Diomus* from Australia, with some species having 11 antennomeres and a few individuals occasionally having only 10 antennomeres (Pang and Ślipiński 2009, Pang and Ślipiński 2010, Vandenberg and Hanson 2019).

*D.guilavoguii* closely resembles *D.hennessyi* Fürsch, 1987 from Zaire, Africa in external appearance and male genitalia. Fürsch (1987) described *D.hennessyi*, based on the specimens collected from Zaire and Nigeria, showing that the penis guide with a horned projection at the proximal apex, especially the peculiar shape of the penis capsule which matches well with the illustration given by Duverger (1994) (Fig. 5). Gordon (1999) provided more detailed descriptions of *D.hennessyi*, based on the examination of a large series of specimens from South America and Africa, including the type series of this species. He also pointed out that *D.hennessyi* is native to South America, but it was introduced into some areas of Africa for biological control of cassava mealybug, *Phenacoccusmanihoti* Matile-Ferrero, 1977 (Gordon 1999). Unfortunately, neither Fürsch (1987) nor Gordon (1999) provided the illustrations of the whole penis. Based on the above mentions, we considered that *D.guilavoguii* may be a synonym of *D.hennessyi*. However, since we have not been able to examine the holotype of *D.hennessyi* deposited in the Musée royal de l'Afrique centrale, Tervuren, Belgium and *D.guilavoguii* housed in the Museum National d’Histoire Naturelle, Paris, France, further confirmation is needed regarding the relationship between these two species.

There have been no deliberate introductions of *D.guilavoguii* in China. When and how did this species spread to Guangdong Province, China? As aforementioned, *P.marginatus* is native to Mexico and Central America. Although this invasive pest has also been reported in Africa, we suspect that *D.guilavoguii* was likely spread to Guangdong, China from Central America along with *P.marginatus*. However, its diffusion path remains to be further studied.

## Supplementary Material

XML Treatment for Diomus

XML Treatment for Diomus guilavoguii
